# Prevalence of on time administration of carbapenem and its impact on PK/PD target attainment in hospitalized patients: a multicenter retrospective study

**DOI:** 10.3389/fcimb.2025.1690269

**Published:** 2025-10-17

**Authors:** Jin Song, Xiaoping Pang, Huadong Chen, Ping Zhang, Miaofa Ying, Renai Xu, Zhenwei Yu

**Affiliations:** ^1^ Nursing Department, Sir Run Run Shaw Hospital, School of Medicine, Zhejiang University, Hangzhou, China; ^2^ Department of Pharmacy, Taizhou Hospital of Zhejiang Province Affiliated to Wenzhou Medical University, Taizhou, China; ^3^ Department of Pharmacy, Affiliated Dongyang Hospital of Wenzhou Medical University, Dongyang, China; ^4^ Department of Pharmacy, Sir Run Run Shaw Hospital, School of Medicine, Zhejiang University, Hangzhou, China; ^5^ Department of Pharmacy, The First Affiliated Hospital of Wenzhou Medical University, Wenzhou, Zhejiang, China; ^6^ Research Center for Clinical Pharmacy, College of Pharmaceutical Science, Zhejiang University, Hangzhou, China

**Keywords:** meropenem, imipenem, administration time, therapeutic drug monitoring, PK/PD

## Abstract

**Objective:**

On time administration of carbapenem is important, but there are few data concerning the prevalence of timeliness and its impact on pharmacokinetic/pharmacodynamic (PK/PD) target achievement in hospitalized patients.

**Methods:**

This was a multicenter retrospective study. Inpatients who received imipenem or meropenem for more than 3 consecutive days were included according to preset criteria. Patient information and dosing information were collected. The actual administration intervals were compared to the scheduled intervals, and those within a 1-hour error interval were defined as standard time window administration (STWA); otherwise, they were defined as noncompliant time window administration (NTWA). The 100% *f*T_>MIC_ and 100% *f*T_>4×MIC_ targets were applied for PK/PD target attainment analysis. A multivariable logistic regression model was used to identify independent risk factors associated with timely administration and the PK/PD target attainment rate.

**Results:**

A total of 474 patients and 1,372 actual administration intervals were included in this study. Among these patients, 82 had drug concentration data and were analyzed for PK/PD target attainment. A total of 427 dosing intervals (31.12%) complied with the standard time window and were STWA, whereas 945 (68.88%) were NTWA. Weekend, night shift, and scheduled dosing intervals were found to be independent influencing factors for STWA. STWA was an independent influencing factor for the 100% *f*T_>MIC_ and 100% *f*T_>4×MIC_ target attainment rates.

**Conclusion:**

Our results indicate a low rate of on time carbapenem administration. The on time carbapenem administration was a risk factor for PK/PD target attainment and should be well controlled in clinical practice.

## 1 Introduction

Carbapenems, mainly meropenem and imipenem–cilastatin, exert rapid bactericidal effects by binding to penicillin-binding proteins (PBPs) and inhibiting bacterial cytoderm synthesis. They have good activity against gram-positive bacteria, gram-negative bacteria, and multidrug-resistant (MDR) pathogens (e.g., extended-spectrum β-lactamase-producing gram-negative bacteria) (Mehta et al., 2021; Gerges et al., 2023). According to pharmacokinetic/pharmacodynamic (PK/PD) principles, carbapenems are classified as time-dependent antibiotics, with clinical efficacy strongly correlated with the time fraction of the free drug concentration exceeding the bacterial minimum inhibitory concentration (MIC) during the dosing interval, expressed as *f*T_>MIC_ (Gatti et al., 2021; Maguigan et al., 2021; Cizmarova et al., 2024). Evidence indicates that achieving *f*T_>MIC_ ≥ 40% serves as the baseline efficacy threshold, whereas critically ill patients require intensified targets of 100% *f*T_>1-4×MIC_ to optimize therapeutic outcomes and mitigate resistance development (Heffernan et al., 2018; Guilhaumou et al., 2019; Beganovic et al., 2021; Eslami et al., 2025). Thus, PK/PD target attainment is crucial for clinical effectiveness. Several previous articles have revealed that continuous infusion of carbapenem, a dosing strategy that can increase the PK/PD index, can significantly improve the clinical outcomes of critically ill patients but has no effect on the outcomes of noncritically ill patients (Phe et al., 2020; Liebchen et al., 2021; Wu et al., 2021; Angelini et al., 2023).

Antibiotic dosing intervals are typically designed on the basis of drug half-life and PK/PD models to maintain the required plasma concentrations or drug exposures (Rodríguez-Gascón et al., 2021). Standard time window administration (STWA) is consistent with absolute deviations between actual and scheduled dosing intervals ≤ 1 hour. The administration time being in accordance with the STWA is considered timely (Loput et al., 2022). Non-timely administered drugs may increase fluctuations in the plasma drug concentration and may lower PK/PD target attainment. However, deviations from scheduled dosing intervals frequently occur in clinical practice because of fluctuations in the nursing workload and individual differences (Blignaut et al., 2017; Schutijser et al., 2018; Martyn et al., 2019; Stolic et al., 2022; Allison Rout et al., 2023). However, this issue is often overlooked, and there are few data concerning the prevalence of on time administration of carbapenem, as well as its impact on PK/PD target attainment.

Therefore, we performed this multicenter retrospective study to assess the patterns of administration timeliness for carbapenems, identify risk factors associated with STWA, and further investigate whether on time administration is an independent risk factor for PK/PD target achievement.

## 2 Methods

### 2.1 Study design and ethical approval

This multicenter retrospective study was performed in 3 tertiary hospitals in China. The study protocol was approved by the ethics committee of Sir Run Run Shaw Hospital, School of Medicine, Zhejiang University (reference number 2025-0136). Informed consent was waived as part of the approval.

### 2.2 Patient inclusion

Patients who were hospitalized at 3 research centers from August 2021 to March 2025 and used carbapenem drugs were randomly selected for inclusion. The exclusion criteria were as follows: (a) aged under 18 years; (b) received carbapenem therapy for ≤ 3 consecutive days; (c) had fewer than two consecutive records of dosing timestamps; (d) received carbapenem for urinary infections; and (e) incomplete or missing key clinical data.

### 2.3 Data collection

The following clinical data of the included patients were extracted from the hospital information system: (a) Demographic data, including patient sex, age, weight (kg), Body Mass Index (BMI, kg/m²), and admission department (ICU or non-ICU). (b) Disease severity indicators: vasoactive agent use. (c) Infection information: infection site and pathogen culture results, if available. (d) Carbapenem treatment regimens: drugs (including imipenem–cilastatin and meropenem), dosages, scheduled dosing intervals and treatment durations. (e) Actual administration time: for patients without carbapenem TDM, the actual administration times of carbapenem on the third day of treatment and the time of the last dose of the previous day were recorded. Thus, two to five consecutive dosing intervals can be calculated. For patients with TDM, the dosing intervals of the day TDM was performed were recorded. To analyze the influence of working time on timely administration, weekends and night shifts (as long as the dosing interval included one administration from 10:00 pm to 8:00 am, it was recorded as a night shift) were also recorded.

For patients with carbapenem TDM, additional information was collected: (a) Paired actual administration timestamps. (b) Continuous renal replacement therapy (CRRT) implementation status. (c) Laboratory biomarkers: trough concentration of target drug, red blood corpuscles (RBCs), total protein (TP), albumin (ALB), total bilirubin (TBil), blood urea nitrogen (BUN), creatinine (Cr), high-sensitivity C-reactive protein (hs-CRP), and procalcitonin (PCT).

### 2.4 Data analysis

The administration time deviation was operationally defined as the absolute deviation between the actual and scheduled dosing intervals. Deviations ≤ 1 hour were categorized as STWA, whereas those exceeding this threshold were classified as nonstandard time window administration (NTWA).

In the analysis of the influence of timely administration on PK/PD target attainment, only patients with delayed administration were classified into the NTWA group, and those with early administration were excluded from the analysis. Through comprehensive data integration of patient demographics, carbapenem regimens, and measured drug concentrations, we evaluated the impact of timely administration on target (100% *f*T_>MIC_ and 100% *f*T_>4×MIC_) attainment rates. For the convenience of statistical analysis, an MIC breakpoint of 2 mg/L was used (Bonnin et al., 2022; Fratoni et al., 2024; Beijer et al., 2025; Luo et al., 2025).

Categorical variables are described as counts and percentages and were tested by the Pearson χ2 test or Fisher’s exact test. Normally distributed variables are described as the means with SDs and were compared via the independent t-test or 1-way analysis of variance, whereas other continuous variables are described as medians with quartiles and were tested via the Wilcoxon Mann–Whitney U test or the Kruskal–Wallis test. To determine factors independently associated with the timeliness of drug administration, variables with *P* < 0.1 in the univariate analysis were analyzed by multivariable logistic regression, and a *P* value < 0.05 was considered significant. Relative risk was estimated via odds ratios (ORs) with corresponding 95% confidence intervals. All the statistical tests were performed via SPSS software (version 23.0).

## 3 Results

### 3.1 Clinical characteristics

**Figure 1:**
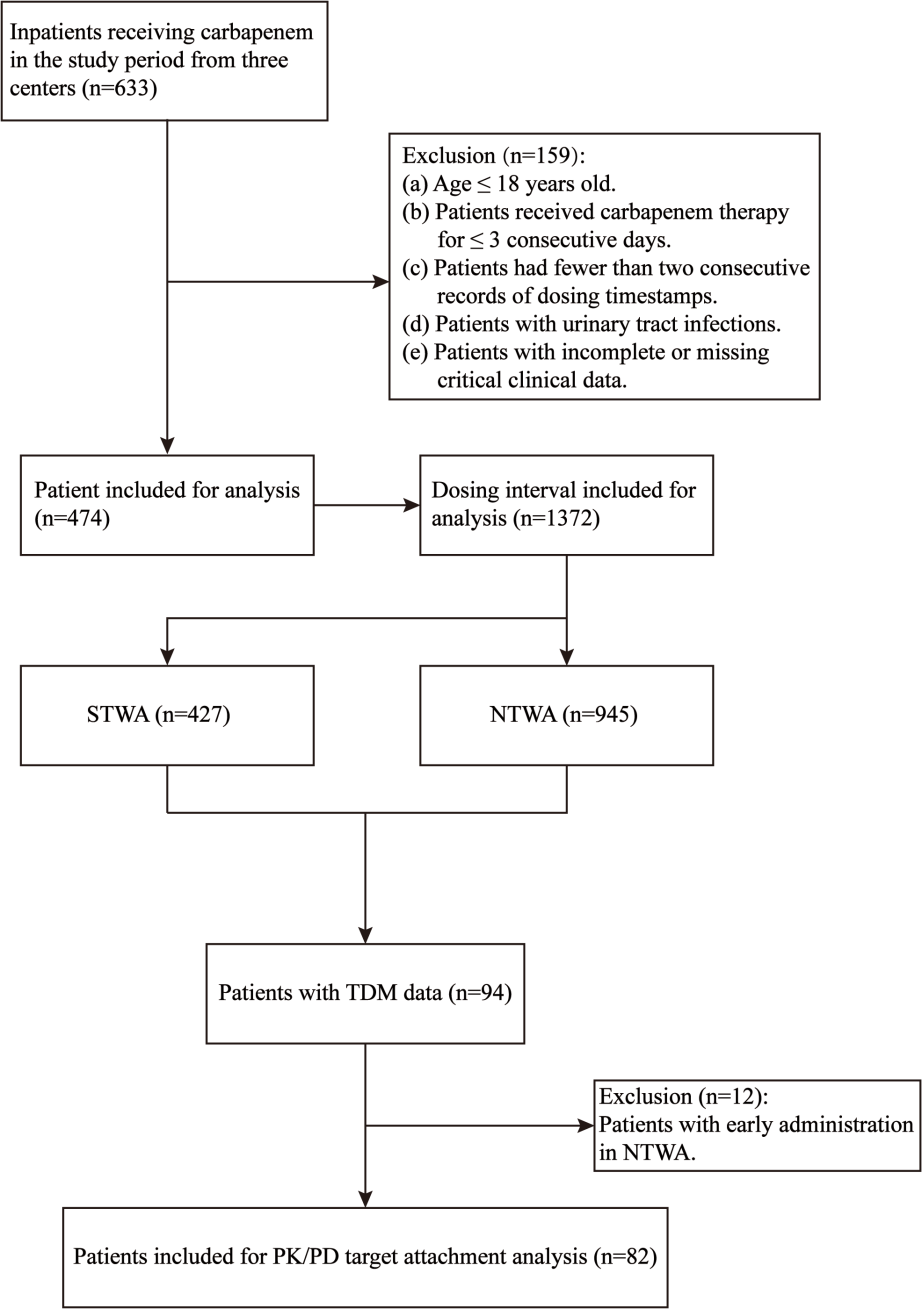
Flowchart of patient exclusion and inclusion.

A total of 633 patients who received carbapenem drug treatment were identified for screening. The inclusion and exclusion processes are shown in **Figure 1**. Finally, 474 patients were eligible for the final analysis and included. The demographic characteristics of the included patients are shown in **Table 1**.

**Table 1:** Demographic characteristics of the included patients.

Variable	Total (n = 474)	Imipenem-cilastatin (n = 268)	Meropenem (n = 206)
Age, Mean ± SD	64.04 ± 17.09	62.60 ± 16.40	65.91 ± 17.82
BMI, Mean ± SD	22.01 ± 4.25	22.13 ± 4.06	21.85 ± 4.50
Sex, n (%)			
Male	335 (70.68)	191 (57.01)	144 (42.99)
Female	139 (29.32)	77 (55.40)	62 (44.60)
Department, n (%)			
Non-ICU	201 (42.41)	137 (68.16)	64 (31.84)
ICU	273 (57.59)	131 (47.99)	142 (52.01)
Vasoactive agent, n (%)	142 (29.96)	54 (38.03)	88 (61.97)
Pulmonary infection, n (%)	174 (36.71)	85 (48.85)	89 (51.15)
Abdominal infection, n (%)	177 (37.34)	123 (69.49)	54 (30.51)
Bloodstream infection, n (%)	56 (11.81)	29 (51.79)	27 (48.21)
Pathogen, n (%)			
Empiric Therapy	236 (49.79)	126 (53.39)	110 (46.61)
Escherichia coli	54 (11.39)	34 (62.96)	20 (37.04)
Klebsiella pneumoniae	70 (14.77)	33 (47.14)	37 (52.86)
Pseudomonas aeruginosa	43 (9.07)	30 (69.77)	13 (30.23)
Acinetobacter baumannii	33 (6.96)	19 (57.58)	14 (42.42)
Scheduled dosing intervals, n (%)			
4 hours	2 (0.42)	0 (0.00)	2 (100.00)
6 hours	64 (13.50)	44 (68.75)	20 (31.25)
8 hours	348 (73.42)	207 (59.48)	141 (40.52)
12 hours	60 (12.66)	17 (28.33)	43 (71.67)
Weekend, n (%)	132 (27.85)	66 (50.00)	66 (50.00)

BMI, Body Mass Index; SD, standard deviation; Non-ICU, non-intensive care unit; ICU, intensive care unit.

### 3.2 Overall distribution of administration timeliness and independent risk factors

As delineated in **Table 2**, the timestamp analysis of 474 patients yielded 1,372 actual administration interval records. The overall rate of on time carbapenem administration was 31.12%. Among all the dosing intervals, 427 intervals (31.12%) complied with the STWA, whereas 945 (68.88%) were noncompliant (NTWA). The details of the STWA and NTWA groups are also shown in **Table 2**. The ICU patients demonstrated a significantly higher STWA compliance rate (35.02%) than did the NTWA patients (*P* = 0.007). Conversely, *Escherichia coli*-infected patients exhibited markedly lower compliance, with compliance rates of only 24.43% in STWA patients and 75.57% in NTWA patients. The 6-hour dosing regimen demonstrated optimal compliance (55.79%), followed by the 12-hour (39.86%) and 4-hour (37.5%) schedules. The 8-hour regimen showed minimal compliance (25.39%). Interestingly, weekend administrations accounted for 37.05% of STWA events versus 62.95% of NTWA events. The same trends were also found for night shifts, which were also different from expectations.

**Table 2:** Prevalence of carbapenem timely administration and multivariate logistic analysis.

Variable	Total (n = 1372)	NTWA (n = 945, 68.88%)	STWA (n = 427, 31.12%)	*P_1_*	*P_2_*	OR (95%CI)
Age, Mean ± SD	62.37 ± 17.09	62.14 ± 16.95	62.89 ± 17.42	0.449		
BMI, Mean ± SD	21.96 ± 4.07	21.82 ± 3.90	22.28 ± 4.43	0.057	0.268	
Sex, n(%)						
Male	959 (69.90)	654 (68.20)	305 (31.80)	0.406		
Female	413 (30.10)	291 (70.46)	122 (29.54)	0.406		
ICU, n(%)	594 (43.29)	386 (64.98)	208 (35.02)	0.007	0.378	
Vasoactive agent, n(%)	233 (16.98)	157 (67.38)	76 (32.62)	0.588		
Pulmonary infection, n(%)	510 (37.17)	348 (68.24)	162 (31.76)	0.693		
Abdominal infection, n(%)	503 (36.66)	340 (67.59)	163 (32.41)	0.435		
Bloodstream infection, n(%)	100 (7.29)	70 (70.00)	30 (30.00)	0.801		
Pathogen, n(%)						
Empiric Therapy	724 (52.77)	493 (68.09)	231 (31.91)	0.508		
*Escherichia coli*	176 (12.83)	133 (75.57)	43 (24.43)	0.041	0.060	
*Klebsiella pneumoniae*	177 (12.90)	119 (67.23)	58 (32.77)	0.612		
*Pseudomonas aeruginosa*	117 (8.53)	83 (70.94)	34 (29.06)	0.615		
*Acinetobacter baumannii*	65 (4.74)	42 (64.62)	23 (35.38)	0.448		
Imipenem-cilastatin, n(%)	830 (60.50)	570 (68.67)	260 (31.33)	0.841		
Meropenem, n(%)	542 (39.50)	375 (69.19)	167 (30.81)	0.841		
Scheduled dosing intervals, n(%)						
4 hours	8 (0.58)	5 (62.50)	3 (37.50)	0.697	0.217	2.62 (0.57 ~ 12.09)
6 hours	190 (13.85)	84 (44.21)	106 (55.79)	<.001	**<.001**	3.55 (2.52 ~ 5.01)
8 hours	1036 (75.51)	773 (74.61)	263 (25.39)	<.001	–	1.00 (Reference)
12 hours	138 (10.06)	83 (60.14)	55 (39.86)	0.02	**<.001**	3.33 (2.15 ~ 5.17)
Weekend, n(%)	359 (26.17)	226 (62.95)	133 (37.05)	0.005	**0.007**	1.47 (1.11 ~ 1.95)
Night shift, n(%)	637 (46.43)	376 (59.03)	261 (40.97)	<.001	**<.001**	3.14 (2.39 ~ 4.12)

BMI, Body Mass Index; SD, standard deviation; ICU, intensive care unit; STWA, standard time window administration; NTWA, non-standard time window administration; OR, Odds Ratio; CI, Confidence Interval; P1, Univariate analysis P-value; P2, Multivariate analysis P-value. The bold P-values indicate statistical significant difference.

The multivariate regression results are also shown in **Table 2**. The analysis revealed that both weekends (OR = 1.47, 95% CI = 1.11–1.95, *P* = 0.007) and night shifts (OR = 3.14, 95% CI = 2.39–4.12, *P* < 0.001) were significantly associated with an increased likelihood of STWA. Furthermore, compared with the 8-hour dosing interval, both the 6-hour dosing interval (OR = 3.55, 95% CI = 2.52–5.01, *P* < 0.001) and the 12-hour dosing interval (OR = 3.33, 95% CI = 2.15–5.17, *P* < 0.001) were also significantly associated with a greater likelihood of STWA (**Table 3**).

**Table 3:** Distribution of 100% *f*T_>MIC_ achievement and multivariate logistic analysis.

Variable	Total (n = 82)	Unachieved (n = 40, 48.8%)	Achieved (n = 42, 51.2%)	*P_1_*	*P_2_*	OR (95%CI)
Age, Mean ± SD	69.21 ± 17.54	62.58 ± 18.47	75.52 ± 14.13	0.002	**0.004**	1.06 (1.02 ~ 1.10)
BMI, Mean ± SD	21.82 ± 4.81	21.85 ± 4.22	21.79 ± 5.40	0.956		
TP, M (Q_1_, Q_3_)	52.10 (47.50, 55.60)	53.05 (48.03, 56.17)	51.30 (47.48, 54.00)	0.238		
ALB, M (Q_1_, Q_3_)	28.80 (27.10, 31.35)	29.35 (27.57, 32.95)	28.30 (27.00, 30.50)	0.178		
RBC, M (Q_1_, Q_3_)	2.90 (2.46, 3.43)	3.12 (2.57, 3.45)	2.88 (2.39, 3.25)	0.192		
TBil, M (Q_1_, Q_3_)	20.35 (11.07, 51.58)	19.90 (9.52, 34.70)	23.60 (12.28, 60.62)	0.319		
BUN, M (Q_1_, Q_3_)	7.91 (5.61, 12.08)	6.75 (4.83, 9.44)	9.93 (6.47, 14.01)	0.03	0.415	
Cr, M (Q_1_, Q_3_)	74.00 (51.00, 106.50)	63.50 (43.75, 82.25)	91.00 (60.75, 125.75)	0.139		
hs-CRP, M (Q_1_, Q_3_)	136.70 (57.10, 190.45)	122.05 (48.07, 158.73)	155.35 (71.22, 215.88)	0.1		
PCT, M (Q_1_, Q_3_)	2.76 (0.38, 22.53)	2.17 (0.44, 17.75)	3.85 (0.36, 34.46)	0.194		
Sex, n (%)						
Male	60 (73.17)	29 (48.33)	31 (51.67)	0.894		
Female	22 (26.83)	11 (50.00)	11 (50.00)	0.894		
Vasoactive agent, n(%)	64 (78.05)	28 (43.75)	36 (56.25)	0.092	0.079	
Pulmonary infection, n(%)	31 (37.80)	15 (48.39)	16 (51.61)	0.956		
Abdominal infection, n(%)	25 (30.49)	14 (56.00)	11 (44.00)	0.388		
Bloodstream infection, n(%)	20 (24.39)	8 (40.00)	12 (60.00)	0.368		
Pathogen, n (%)						
Empiric Therapy	28 (34.15)	14 (50.00)	14 (50.00)	0.874		
*Escherichia coli*	7 (8.54)	5 (71.43)	2 (28.57)	0.227		
*Klebsiella pneumoniae*	24 (29.27)	13 (54.17)	11 (45.83)	0.531		
*Pseudomonas aeruginosa*	14 (17.07)	6 (42.86)	8 (57.14)	0.627		
*Acinetobacter baumannii*	11 (13.41)	2 (18.18)	9 (81.82)	0.044	**0.045**	6.11 (1.04 ~ 35.88)
Scheduled dosing intervals, n (%)						
6 hours	14 (17.07)	7 (50.00)	7 (50.00)	0.92		
8 hours	54 (65.85)	29 (53.70)	25 (46.30)	0.47		
12 hours	14 (17.07)	4 (28.57)	10 (71.43)	0.106		
STWA, n(%)	35 (42.68)	12 (34.29)	23 (65.71)	0.025	**0.023**	3.66 (1.20 ~ 11.16)
CRRT, n(%)	13 (15.85)	3 (23.08)	10 (76.92)	0.054	0.232	

BMI, Body Mass Index; SD, standard deviation; STWA, standard time window administration; NTWA, non-standard time window administration; TP, total protein; ALB, albumin; RBC, red blood corpuscles; TBil, total bilirubin; BUN, blood urea nitrogen; Cr, creatinine; hs-CRP, high-sensitivity C-reactive protein; PCT, procalcitonin; CRRT, continuous renal replacement therapy; OR, Odds Ratio; CI, Confidence Interval; P1, Univariate analysis P-value; P2, Multivariate analysis P-value. The bold P-values indicate statistical significant difference.

### 3.3 PK/PD target attainment rate and independent risk factors

**Figure 2:**
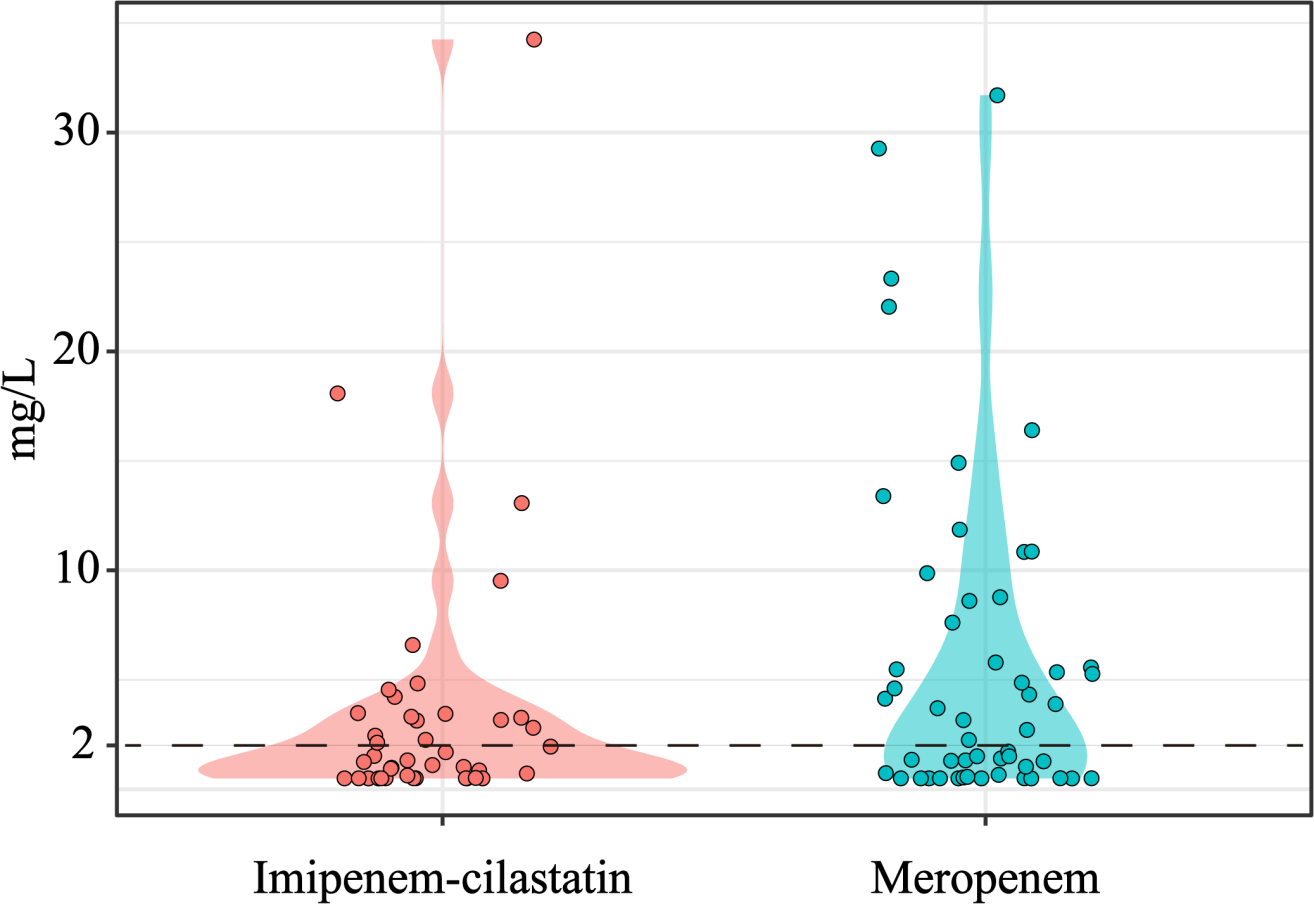
Plasma concentration distributions of imipenem and meropenem.

Finally, 82 patients with TDM results were analyzed for PK/PD target attainment. The distributions of meropenem and imipenem concentrations are shown in **Figure 2**. Among these patients, 42 (51.2%) achieved the PK/PD target of 100% *f*T_>MIC_, whereas 16 (19.5%) attained the stricter target of 100% *f*T_>4×MIC_ (**Table 3**; **Supplementary Table S1**). Univariate analysis revealed that patients who achieved 100% *f*T_>MIC_ were significantly older than unachievers (75.52 ± 14.13 vs. 62.58 ± 18.47 years, *P* < 0.05) and presented higher median BUN levels [9.93 (IQR 6.47–14.01) vs. 6.75 (IQR 4.83–9.44), *P* < 0.01]. Notably, compliance with the STWA was significantly greater in the 100% *f*T_>MIC_ group than in the unachieved group (65.71% vs. 34.29%, *P* = 0.025). However, the STWA compliance rates are markedly lower for the 100% *f*T_>4×MIC_ target (34.29% vs. 65.71%, *P* = 0.006).

Multivariate logistic regression incorporating age, BUN, *Acinetobacter baumannii* infection, vasoactive agent, STWA, and CRRT. The results indicated that age (OR = 1.06, 95% CI = 1.02–1.10, *P* = 0.004), *Acinetobacter baumannii* infection (OR = 6.11, 95% CI = 1.04–35.88, *P* = 0.045), and STWA (OR = 3.66, 95% CI = 1.20–11.16, *P* = 0.023) were independent risk factors associated with 100% *f*T_>MIC_ target attainment (**Table 3**). Notably, STWA was also positively correlated with 100% *f*T_>4×MIC_ attainment (**Supplementary Table S1**).

## 4 Discussion

To the best of our knowledge, this is the first multicenter study that evaluated the prevalence of on time administration of carbapenems and its impact on PK/PD target attainment in hospitalized patients. A total of 474 patients and 1,372 dosing intervals from three tertiary hospitals were included in this study, which made the results representative. Notably, the prevalence of STWA is low. Scheduled dosing intervals were identified as independent risk factors for STWA, which should be well controlled in clinical practice. Furthermore, STWA was an independent risk factor associated with PK/PD target attainment, which emphasized the importance of on time administration.

Our study evaluated the timeliness of carbapenem administration in hospitalized patients. Notably, only 31.12% of the carbapenems were administered within the STWA, which was lower than the available prevalence rates. Tolley and Poon reported that the probabilities of timing errors occurring in hospitals were 11.5% and 4.77%, respectively (Poon et al., 2010; Tolley et al., 2022). For time-critical medications, the incidence of timing errors in administration is greater. Craig et al. reported a 68.88% adherence rate to the STWA for time-critical medications (Furnish et al., 2021). Our study demonstrated a significantly lower rate of timely carbapenem administration. This discrepancy likely reflects the confluence of several factors, notably the inherently time-consuming preparation process specific to carbapenems and potential inefficiencies within the hospital’s medication management workflow (encompassing physician order entry, pharmacy dispensing, and nursing execution) (Yu et al., 2023). Multivariate regression analysis revealed that administration during weekends and night shifts was positively correlated with STWA attainment. This counterintuitive observation may be attributed to reduced competing clinical demands (e.g., fewer scheduled surgeries or physician rounds) during these periods, potentially enabling nursing staff to prioritize medication administration tasks. Compared with the 8-hour dosing interval, both the 6-hour and 12-hour intervals were significantly associated with a greater likelihood of STWA. This association may be attributed to the better alignment of the 6-hour dosing interval with routine nursing workflows and shift handovers, facilitating integration into planned care activities. The 12-hour intervals, typically administered at fixed times (e.g., 8:00 am and 8:00 pm), benefit from a simpler, twice-daily regimen that is less prone to omission or confusion. Importantly, the 8-hour dosing interval constituted the predominant group in this study cohort, whereas data for the 6-hour dosing interval and 12-hour dosing interval were comparatively limited. This imbalance in sample sizes may introduce potential bias into the observed associations.

These findings highlight systemic inefficiencies in routine clinical workflows and emphasize the urgent need for staffing optimization during weekdays and daytime shifts. In addition, previous studies indicate that communication between medical teams may be an important factor in patient care, and the assessment ability of healthcare workers and awareness of time-critical drugs should be improved (Vijayan et al., 2021; Amano et al., 2023; Wu et al., 2025). To increase carbapenem stewardship, hospitals should implement targeted interventions: (1) standardize workflow protocols to minimize interruptions during peak hours; (2) integrate automated alerts for high-priority antimicrobials into electronic health records; and (3) conduct regular staff training to reinforce the clinical significance of timely carbapenem administration.

While pre-clinical investigations have historically informed PK/PD targets for beta-lactam antibiotics, the applicability of these thresholds requires careful contextualization. This value originated largely from animal infection models, where carbapenems demonstrated efficacy at 40% *f*T_>MIC_ owing to their prolonged post-antibiotic effect (Assefa et al., 2024). However, such thresholds may not directly translate to clinical practice, especially in critically ill patients who exhibit altered pharmacokinetics due to underlying illness, comorbidities, or life-saving treatment modalities. In these populations, targeting higher thresholds such as 100% *f*T_>MIC_ or even 100% *f*T_>4×MIC_ is increasingly adopted (De Waele et al., 2013; Abdulla et al., 2020; Peng et al., 2025; Tseng et al., 2025). These more aggressive targets aim to compensate for pharmacokinetic variability and ensure adequate drug exposure throughout the dosing interval, particularly when treating pathogens with elevated MICs or in immunocompromised hosts.

In the present study, TDM results were available for 82 patients. The target of 100% *f*T_>MIC_ was achieved in 51.2% of patients, whereas 19.5% attained 100% *f*T_>4×MIC_. In a study investigating the relationship between % *f*T_>MIC_ and the clinical efficacy of meropenem, the number of sepsis patients treated with meropenem reached 40% *f*T_>MIC_ and 100% *f*T_>MIC_, which were 92.9% and 71.4%, respectively (Vijayan et al., 2021). *Kim* et al. achieved a 30% compliance rate of 100% *f*T_> 4×MIC_ by administering 2 g of meropenem every 6 hours as a 3 h extended infusion (Kim et al., 2024). This discrepancy may be attributable to methodological differences between the studies. Specifically, *Kim* et al. applied a lower MIC threshold (1 mg/L for meropenem), in contrast to the 2 mg/L threshold used in our study. *Areskog* et al. demonstrated that 88% of meropenem-treated patients achieved 100% *f*T_>MIC_ and that 53% reached 100% *f*T_>4×MIC_ (Areskog Lejbman et al., 2024). Notably, their protocol employed a standardized regimen of 1 g every 8 hours, whereas our study included individualized dosing regimens (0.5 g or 1 g administered every 6, 8, or 12 hours), which likely contributed to the observed pharmacokinetic variability.

Timely administration critically influences the PK profiles of time-dependent antimicrobial agents. A systematic analysis of ICU patients receiving carbapenems revealed PK/PD target attainment rates, with 100% *f*T_>MIC_ achieved in 65.71% of cases and 100% *f*T_>4×MIC_ achieved in 34.29% of cases. Multivariate regression analysis demonstrated that timely administration significantly improved both the 100% *f*T_>MIC_ and 100% *f*T_>4×MIC_ attainment rates. Carbapenems exhibit time-dependent bactericidal activity, where clinical efficacy is maximized when *f*T_>MIC_ exceeds a certain threshold. For carbapenems, maintaining *f*T_>MIC_ ≥ 40% of the dosing interval is critical; achieving 100% *f*T_>MIC_ further optimizes bacterial eradication and suppresses resistance (Tilanus and Drusano, 2023; Pokorná et al., 2024). For short-half-life β-lactams (e.g., meropenem, imipenem–cilastatin), delays cause a rapid decline in serum concentrations, which would be below the MIC threshold. Timely administration ensures consistent concentration–time curve (AUC) overlap, sustaining drug levels above the MIC throughout the interval. Pathogens such as *Pseudomonas aeruginosa* exhibit adaptive resistance when exposed to subinhibitory antibiotic concentrations. Delayed dosing creates sub-MIC windows, allowing bacterial regrowth and increasing the risk of resistance mutations (Chen et al., 2021; Sanz-García et al., 2022). Most previous studies have focused on the rate of attaining PK/PD targets for carbapenems with continuous and intermittent infusions (Wunderink et al., 2021; Monti et al., 2023; Johnson et al., 2024). Our findings underscore that strict compliance with scheduled dosing intervals ensures sustained drug concentrations above the MIC and 4×MIC thresholds, thereby maximizing bactericidal efficacy and mitigating resistance selection.

The clinical imperative for on time administration is magnified in critically ill patients. ICU patients frequently exhibit altered pharmacokinetics due to pathophysiological changes or extracorporeal therapies, leading to unpredictable drug exposure (Morales Castro et al., 2023). Timely administration mitigates concentration fluctuations, preventing transient trough concentrations below the MIC (even for 1–2 hours), so that it blocks the selective proliferation of drug-resistant mutants. To operationalize precision dosing for time-critical medications, hospitals must implement workflow innovations. For carbapenems, strategies such as premixed formulations, smart infusion pumps, and rapid bedside administration systems can minimize delays between prescription and drug delivery.

This study has several limitations inherent to its retrospective design, which may introduce selection bias and unmeasured confounding factors. The analysis did not directly assess the associations between administration timeliness and clinical outcomes such as mortality or resistance emergence. Future prospective cohort studies are warranted to establish causality between timely administration and therapeutic efficacy. Despite the overall large sample size of this study, only 82 patients (17.3%) had TDM data available for PK/PD analysis. This may introduce selection bias, as patients with more complex conditions are more likely to undergo TDM monitoring. Therefore, the findings still require further validation with larger datasets. An international survey revealed considerable variation in TDM practices for β-lactam antibiotics in intensive care units (Wong et al., 2014). Although carbapenem TDM has been reported in clinical settings, most studies focus on meropenem (Joynt et al., 2023). Moreover, a literature search using the keywords “China” AND “carbapenem” AND “TDM” yielded very few results, indicating that carbapenem TDM has not yet been routinely implemented in clinical practice in China. This limitation underscores the need for more prospective studies on carbapenem TDM in the future.

Additionally, further research should address critical knowledge gaps: (1) the development of dose-compensation protocols for delayed administration and (2) the clinical validation of novel delivery technologies such as nanoparticle-based controlled-release systems to bridge the gap between theoretical PK/PD targets and real-world effectiveness.

## 5 Conclusions

This multicenter retrospective study investigated the prevalence of on time carbapenem administration, and the results indicated that the timeliness of carbapenem administration was poor. Several factors, including scheduled dosing intervals, weekend days and night shifts, were significantly associated with improved timely administration. Furthermore, on time administration was found to be associated with PK/PD target attainment rates (100% *f*T_>MIC_ and 100% *f*T_>4×MIC_), which indicated that timely administration might be important in optimizing the efficacy of antibiotics. In the future, systematic improvement and interdisciplinary collaboration are needed to improve the timeliness of drug administration.

## Data Availability

The original contributions presented in the study are included in the article/**Supplementary Material**. Further inquiries can be directed to the corresponding authors.

## Supplementary material

Supplementary Table 1 Distribution of 100% *f*T_>4×MIC_ achievement and multivariate logistic analysis.
