# Supplementary material for: Prevalence of on time administration of carbapenem and its impact on PK/PD target attainment in hospitalized patients: a multicenter retrospective study
Source: Front Cell Infect Microbiol. 2025 Oct 17;15:1690269. doi: 10.3389/fcimb.2025.1690269 (PMC12575351; doi:10.3389/fcimb.2025.1690269)
Supplement: Supplementary Table 1 — Distribution of 100% fT>4×MIC achievement and multivariate logistic analysis. [file DataSheet1.docx]

**Table S1** Distribution of 100% *f*T_>4×MIC_ achievement and multivariate logistic analysis.

|  | **Total**  **(n = 82)** | **Unachieved**  **(n = 66)** | **Achieved**  **(n = 16)** | ***P1*** | ***P2*** | **OR (95%CI)** |
| --- | --- | --- | --- | --- | --- | --- |
| **Age, Mean ± SD** | 69.21 ± 17.54 | 67.44 ± 17.45 | 76.50 ± 16.49 | 0.071 | 0.471 |  |
| **BMI, Mean ± SD** | 21.82 ± 4.81 | 21.91 ± 4.83 | 21.46 ± 4.89 | 0.745 |  |  |
| **TP, M (Q₁, Q₃)** | 52.10 (47.50, 55.60) | 52.10 (48.10, 55.40) | 51.30 (45.15, 56.35) | 0.425 |  |  |
| **ALB, M (Q₁, Q₃)** | 28.80 (27.10, 31.35) | 28.90 (27.00, 31.72) | 28.20 (27.35, 30.50) | 0.536 |  |  |
| **RBC, M (Q₁, Q₃)** | 2.90 (2.46, 3.43) | 2.92 (2.48, 3.45) | 2.63 (2.36, 3.11) | 0.523 |  |  |
| **TBil, M (Q₁, Q₃)** | 20.35 (11.07, 51.58) | 20.20 (10.70, 47.70) | 28.50 (13.35, 52.70) | 0.942 |  |  |
| **BUN, M (Q₁, Q₃)** | 7.91 (5.61, 12.08) | 7.75 (4.98, 11.74) | 10.69 (6.36, 21.19) | 0.038 | 0.351 |  |
| **Cr, M (Q₁, Q₃)** | 74.0 (51.0, 106.5) | 68.0 (47.0, 90.7) | 112.0 (91.2, 131.2) | 0.172 |  |  |
| **hs-CRP, M (Q₁, Q₃)** | 136.7 (57.1, 190.4) | 125.1 (56.9, 189.0) | 166.7 (86.9, 206.5) | 0.269 |  |  |
| **PCT, M (Q₁, Q₃)** | 2.76 (0.38, 22.5) | 2.07 (0.32, 19.1) | 17.6 (0.53, 32.9) | 0.335 |  |  |
| **Sex, n(%)** |  |  |  |  |  |  |
| Male | 60 (73.17) | 49 (81.67) | 11 (18.33) | 0.657 |  |  |
| Female | 22 (26.83) | 17 (77.27) | 5 (22.73) | 0.657 |  |  |
| **Vasoactive agent, n(%)** | 64 (78.05) | 52 (81.25) | 12 (18.75) | 0.743 |  |  |
| **Pulmonary infection, n(%)** | 31 (37.80) | 26 (83.87) | 5 (16.13) | 0.548 |  |  |
| **Abdominal infection, n(%)** | 25 (30.49) | 24 (96.00) | 1 (4.00) | 0.043 | 0.204 |  |
| **Bloodstream infection, n(%)** | 20 (24.39) | 14 (70.00) | 6 (30.00) | 0.18 |  |  |
| **Pathogen, n(%)** |  |  |  |  |  |  |
| Empiric Therapy | 28 (34.15) | 19 (67.86) | 9 (32.14) | 0.043 | 0.502 |  |
| Escherichia coli | 7 (8.54) | 6 (85.71) | 1 (14.29) | 0.717 |  |  |
| Klebsiella pneumoniae | 24 (29.27) | 21 (87.50) | 3 (12.50) | 0.309 |  |  |
| Pseudomonas aeruginosa | 14 (17.07) | 12 (85.71) | 2 (14.29) | 0.59 |  |  |
| Acinetobacter baumannii | 11 (13.41) | 9 (81.82) | 2 (18.18) | 0.905 |  |  |
| **Scheduled dosing intervals, n(%)** | |  |  |  |  |  |
| 6 hours | 14 (17.07) | 12 (85.71) | 2 (14.29) | 0.59 | 0.600 |  |
| 8 hours | 54 (65.85) | 47 (87.04) | 7 (12.96) | 0.043 | Ref |  |
| 12 hours | 14 (17.07) | 7 (50.00) | 7 (50.00) | 0.003 | 0.220 |  |
| **Night shift, n(%)** | 64 (78.05) | 53 (82.81) | 11 (17.19) | 0.321 |  |  |
| **STWA, n(%)** | 35 (42.68) | 23 (65.71) | 12 (34.29) | 0.006 | **0.023** | 5.09 (1.25 ~ 20.74) |
| **CRRT, n(%)** | 13 (15.85) | 9 (69.23) | 4 (30.77) | 0.272 |  |  |

SD: standard deviation; Non-ICU: non-intensive care unit; ICU: intensive care unit; STWA: standard time window administration; NTWA: non-standard time window administration; TP: total protein; ALB: albumin; RBC: red blood corpuscles; Tbil: total bilirubin; Cr: creatinine; hs-CRP: high-sensitivity C-reactive protein; PCT: procalcitonin; CRRT: continuous renal replacement therapy. P1: P value for univariate analysis; P2: P value for multivariate logistic regression; OR: Odds Ratio, CI: Confidence Interval.
